# Micro- and small-scale enterprises’ financing preference in line with POH and access to credit: empirical evidence from entrepreneurs in Ethiopia

**DOI:** 10.1186/s13731-022-00246-z

**Published:** 2022-10-21

**Authors:** Hayelom Abrha Meressa

**Affiliations:** Assosa University, Assosa, Ethiopia

**Keywords:** Assosa Zone, Credit, Financing, Logistic regression, Micro- and small enterprises

## Abstract

The purpose of this study was to examine factors that determine micro- and small-scale enterprises’ financing preference in line with pecking order theory and access to credit in Benishangul-Gumuz Regional State of Ethiopia. The study used primary data collected using cross-sectional survey questionnaire. The sample of this study was 296 enterprises selected using proportional stratified random sampling technique. The data were analyzed using descriptive and logistic regression analysis. The results of probit estimation revealed that business experience, collateral, gender, motivation and enterprises’ sectoral engagement affect financing preference. To investigate access to credit determinants, only enterprises that need to raise capital through credit were considered. The empirical results, therefore, revealed that business experience, size, sectoral engagement, collateral, interest rate, loan repayment period, and preparation of business plan, financial reporting, location and educational background of entrepreneurs affect access to credit of enterprises. Before any generalization of the results can be made, more evidence is needed on enterprises’ financing preference and access to credit determinants for the fact that the empirical tests were conducted only on 296 entrepreneurs since 2019. Therefore, the findings are valid and practicable only for the entrepreneurs under the study and the results cannot be assumed to extend beyond this group of entrepreneurs to different study periods. The study makes an original contribution to the literature of small business finance by investigating determinants of micro- and small-scale enterprises’ financing preference and access to credit in Benishangul Gumuz Regional State of Ethiopia as a developing country.

## Introduction

In spite of their contribution to employment, micro- and small-scale enterprises (MSSEs, hereafter) face various constraints while operating their business (Ali et al., 2016; Belanova, 2013; Daksa et al., 2018; Gupta et al., 2013; Haron et al., 2013; Nguyen et al., 2015; Rossi et al., 2016). However, the difficulty of financing is a universal problem to the sector and the issue is sever in developing countries (Abor & Biekpe, 2009; Sahiti & Smith, 2017; Wu et al., 2008). As a developing country, access to finance is by far the most critical bottleneck of MSSEs in Ethiopia (Assefa et al., 2014; Menkir, 2016; Tarfasa et al., 2016). In order to promote MSSEs as engines of growth, however, it is essential to understand their financing decision in general and credit market participation interest in particular as well as the bottlenecks surrounding their access to external credit (Mersha & Ayenew, 2017). Otherwise, the financial constraints they face in their operations will daunt their development negatively that could limit their potential to drive the national economy as expected (Manaye & Tigro, 2017). Therefore, it is indispensable to study financing decision of small business in developing countries like Ethiopia, where the financial market is weakly efficient with high information asymmetry between financial institutions and MSSEs (Gebru, 2009).

Globally, the financing decision of small businesses has important implications for their performance, ability to succeed, risk of failure and potential for future development (Ahmad & Atniesha, 2018). In this regard, though there are many competing theories of financing decisions of firms following the work of Modigliani & Miller, the existing literature emphasizes that the financing theory of large firms has very limited applicability to SMEs except static trade-off theory and pecking order theory (Daskalakis & Schizas, 2013; Johnsen & McMahon, 2005). According to the static trade of theory, financing decision is carried out based on the cost–benefit evaluation with respect to the use of debt to finance operations. In other words, the theory hypothesizes that firms balance the tax advantages obtained by debt against the probabilities of bankruptcy (Myers, 1984). In this regard, micro-entrepreneurs are required to have substantial reliable data. However, MSEs usually have weakly organized accounting system adding complexity to data generation problems which makes its applicability to the sector difficult (Gebru, 2009). On the other hand, pecking order theory of financing is driven by information asymmetry affecting financing behavior and it hypothesizes that the cost of financing increases with asymmetric information, and firms prefer internal sources over external finance due to adverse selection. When outside funds are necessary, firms prefer debt to equity because of lower information costs associated with debt issues focusing on the relationship between information asymmetry and financing options. (Myers & Majiuf, 1984; Osei-Assibey et al., 2012). Therefore, the POH has a relatively dominant explanation for small business financing over tradeoff as long as information asymmetry, which underpins the POH, is greater in in small firms (Paul et al., 2007). In this regard, the understanding of financing preference determinants of small business is important to allow the application of correct measures to encourage the availability of capital to the sector (Serrasqueiro & Caetano, 2015). In line with this, few studies were carried out in different countries to investigate the determinants of financing preference of micro- and small enterprises and test whether there is evidence of hierarchical preference ordering as predicted by pecking order theory though the findings are not consistent (Daskalakis & Schizas, 2013; Gebru, 2009; Kuruppu & Azeez, 2016; Osei-Assibey et al., 2012; Paul et al., 2007). However, the empirical evidences suggest that generalizing about financing decision issues of SMEs is very difficult because there is difference in defining size the sector, nature of firms, external environment and context diversity of countries (Daskalakis & Schizas, 2013). Therefore, the need to look at the issue from the perspective of developing economies SMEs’ financing decisions still remain unexplored for the fact there are many differences in institutional arrangements and financial markets between developed and developing countries as well as differences in information diffusion of small businesses (Abor & Biekpe, 2009; Rao et al., 2019) and Ethiopia is inclusive.

The decision of choice of financing depends on preference of the owners and possibility and finance accessibility in the financial system (Kuruppu & Azeez, 2016). Therefore, the argument that probability of accessing external finance depends on willfully participation of firms in the credit market and differentiating truly credit constrained firms from non-constrained firms is important while studying access to external credit constraining factors. In this regard, a study on manufacturing enterprises in Amhara Regional State of Ethiopia revealed that most of the enterprises did not seek to obtain external financing in the first place due to many reason (Melesse, 2019) though the main concern of the study was not to test POH in the first place. In most developing countries where access to formal finance by MSEs is woefully limited, it is difficult to tell whether a particular financing pattern is just an issue of preference or desperation borne out from limited access or constraints to formal finance (Osei-Assibey et al., 2012). In Ethiopia, as a developing country with limited financial market, micro-finance institutions(MFIs, hereafter) are suggested to be the providers of credit services to MSEs as indicated in MSSEs’ policy and strategy (MoUDH, 2016) which is confirmed by empirical evidences (Engida et al., 2017; Melesse, 2019). However, many of the institutions may not meet the capital requirements of SMEs especially when the latter grows in size and operation as the long as the institutions themselves are in limited finance (Nega & Hussein, 2016). Moreover, the worst limitation is in MFIs of Benishangul Gumuz Regional State where the small enterprises in the region get serious difficulty of access to credit (Abara & Banti, 2017) and take the lowest percentage share of credit from the total amount of loan provided by micro-finance sector of the country (NBE, 2019).

In literature of small business, there are various factors that may influence access to finance. Some argue that the fundamental reasons behind MSSEs’ constraints of access to credit can be found in their peculiar characteristics, while others argue that MSSEs suffer from financing gaps because of supply side factors (Mazanai & Fatoki, 2012; Nega & Hussein, 2016). Moreover, MSSEs may face financing gaps probably because of combination of factors originating from both the supply and demand sides (Stijn & Tzioumis, 2006). In line with this, systematic review was carried out on studies made by Gebru (2009); Zarook et al., 2013; Nkuah et al., 2013; Gamage, 2013; Haron et al., 2013; Alhassan & Sakara, 2014; Marwa, 2014) Awlachew & Motumma, 2017; Elly & Kaijage, 2017; Fufa, 2016; Kebede et al., 2014; Kuruppu & Azeez, 2016; Makina et al., 2015; Manaye & Tigro, 2017; Mashenene, 2015; Mersha & Ayenew, 2017; Mole & Namusonge, 2016; Nguyen, 2016; Nega & Hussein, 2016; Nguyen et al., 2015; Osano & Languitone, 2016; Waari & Mwangi, 2015 to identify literature-based determinants in which the detail review is discussed in the literature part. The literature review, however, provides inconclusive evidence with regard to the determinants. Moreover, the issue of financial inclusion remains as relevant as ever before (Melesse, 2019) which needs detailed investigation. Therefore, due to the mixed evidence from previous studies and relevance of the issue, the twofold purpose of this study was to examine factors that determine micro- and small-scale enterprises’ financing preference to establish whether these enterprises had followed the pecking order hypothesis in the first place and to investigate access to credit determinants in in Benishangul-Gumuz Regional State of Ethiopia using binary logistic regression.

Against the above background, the present study contributes to the existing body of knowledge in a number of ways. First, unlike the studies in the existing literature focused on access to credit of enterprises ignoring their preference of financing, this research sought to explore whether small businesses had followed the POH while making financing decision in the first place and investigate access to external credit determinants focusing only on entrepreneurs with demand of credit market participation to look at the true constraints. Second, the study is based on cross-sectional data collected from small business owners from Benishangul Gumuz Regional State of Ethiopia to minimize the problem of pooling observations from structurally different economies (Melesse, 2019) as long as differences in institutional arrangements between economies actually merit the need to look at the issue from different perspectives (Abor & Biekpe, 2009). Third, nothing is said about access to credit determinants of small enterprises in the region where there is serious difficulty of enterprises’ access to credit (Abara & Banti, 2017) and the region’s small business sector take the lowest percentage share of credit from the total amount of loan provided by micro-finance sector of the country (NBE, 2019). Moreover, the paper examines the possibility of enterprise size being endogenous to access to credit using two-step IV-probit and sample-induced endogeneity possibility of access to finance using Heckman selection model which has not been addressed yet to the best of the researcher’s knowledge.

The remainder of this paper is structured as follows: “Literature review” section is about review of related literature. “Research methodology” section discusses about research methodology followed by “Empirical results and discussion” section that presents empirical results and discussion. Finally, “Conclusion, implication and direction for future research” section provides the conclusion thereafter forwards implication and direction for future research.

## Literature review

This section presents literature via reviewing works of different researchers on determinants of financing preference and access to credit of micro and small business. Globally, micro- and small business play indispensable role in a nation’s economy. Given that small firms have become increasingly important component of economic development and the role of finance has been viewed as a critical element for development of the sector, it is important to understand the factors that influence their financing decision. With regard to small business, although there is no universally accepted definition, micro enterprises are firms with employees of 1 to 5 and total capital not exceeding birr 50,000 for service sector and birr 100,000 excluding land and building for industries in Ethiopia. Besides, small enterprises are entities that have employees from 6 to 30 with the total capital that ranges between 50,000 to 500,000 birr for services and 100,000 to 1.5 million Birr for industries (MoUDH, 2016). The decision of financing is the foremost critical area for any entrepreneur to start or raise business. Whether they choose traditional route or try something creative, there are many external and internal sources to finance small business (Al-Afifi, 2019; Mittal & Raman, 2020). In this regard, to identify the most commonly used variables as financing preference and access to credit determinants, concentrated and careful systematic review of literature was carried out on relatively recent empirical studies. Accordingly, the review on the determinants is discussed below.

According to the pecking order theory, firms with more fixed assets can easily access secured debt since tangible assets are used as collateral for debt (Vijayakumaran & Vijayakumaran, 2018). Micro- and small enterprises with greater fixed asset have better chance to access credit from formal financial institutions than their counter parts. This is for the reason that fixed asset acts as a screening device and reduces the risk of lending for financial institutions (Kebede et al., 2014). Financial institutions are more likely to approve loans to firms that are able to provide collateral. Due to the existence of asymmetric information, formal financial institutions base their lending decisions on the amount of fixed asset available. Collateral acts as a screening/rationing device and reduces the risk of lending for financial institutions. Small firms are disadvantaged in this regard, due to the fact that they lack collateral security and also they lack a proven credit track record (Osebo, 2014). These institutions demand collateral security to make credit to reduce default of repayment by MSSEs (Mole & Namusonge, 2016). Collateral requirements influence access to finance by small businesses. It is evident that most small businesses are denied and discriminated by the lenders in provision of financing. This is because of high risk and for not having adequate resources to provide as collateral (Osano & Languitone, 2016). Lack of collateral, which may be provided as a security lowers access to finance by MSSEs (Borji & Gashu, 2015). In most financial institutions, to finance MSSEs and to accept loan proposals, the collateral must be 100% or more, equal to the amount of credit extension or finance product (Osano & Languitone, 2016). Thus, low value of collateral reduces access to finance by small business (Mashenene, 2015). In most scenarios, high collateral requirements are a binding constraint for smaller firms since the most common type of collateral used are land and buildings or personal assets. As elsewhere, in developing economies, Ethiopian banks prefer immovable collateral such as land rather than movable assets such as machinery (World Bank Group, 2015). As the provision of collateral plays an indispensable role in easing MSSEs access to debt finance, MSSEs that have more fixed assets tend to utilize higher financial leverage. The reason for this is that these firms can borrow at lower interest rates as their loans are secured with these assets serving as collateral (Mersha & Ayenew, 2017).

Gender of entrepreneurs also plays a significant role in behavior of humans in financing decisions. Females rely more on personal savings to finance their business (Kuruppu & Azeez, 2016). Besides, it has a significant effect on credit access of small business owners. On the one side, being a male owner increases the probability of obtaining loan. On the other side, female owned MSSEs are more credit constraint (Nguyen et al., 2015). It is also evidenced that business owner-manager’s gender is not a significant factor in explaining a small and medium firms’ financing decision (Eniola, 2018). The pecking order theory also suggests that, bigger firms are more likely to use less debt due to lower asymmetric information problems between insiders and outside investors (i.e., larger firms provide more information to lenders than smaller firms, so the cost of issuing new equity is lower than the debt issuing cost) (Vijayakumaran & Vijayakumaran, 2018). Financial institutions are more likely to approve loans to firms that are relative better in terms of capital and number members implying that lending institutions and informal lenders base their lending decisions on the size of the firm/MSSEs (Fufa, 2016).

In terms of sector, firms in the service sector have lower probability to borrow as compared to industry (Nguyen et al., 2015). Business sector in which a firm operates also explain financial decisions of entrepreneurs (Mersha & Ayenew, 2017). Interest rate is also considered as one determinant factor of access to credit. The rate of interest charged on credit determines the cost of credit. The cost credit is the amount the borrower is obligated to pay above the principal sum of the money lent. High interest rates increase the cost of credit. High interest rates on credit may discourage MSSEs from borrowing reducing the accessibility of credit among them (Mole & Namusonge, 2016). There is a negative relationship between high interest rate and access to debt financing from financial institutions by MSSEs. Hence, the higher the interest rate, the lower access to finance and vice versa (Mashenene, 2015). Keeping the above, owners prefer internal source for not paying higher interest. Following from the law of demand and supply, the higher the interest rate, the lower the level of intermediation will be. MSSEs have less debt financing because of high interest rates charged by financial institutions.

In the context of MSSEs requiring extra capital to grow, regular financial reports can provide indications on their ability to produce steady cash flows and to service debt (Mersha & Ayenew, 2017). When lending is based on data supplied through financial statements, firms that cannot produce independently vouched statements are denied credit. Hence, producing financial statements enhances the chance to secure bank credit. In fact, banks require financial statements as part of a loan application (Fanta, 2015). The geographical area where a firm is located in the proximity of banks or MFIs is also believed to have an influence on the firm’s ability to gain external finance. For example, MSEs located outside major cities face greater difficulties in acquiring external finance, especially long-term debt, compared with their counterparts operating in cities (Mersha & Ayenew, 2017). SMEs located in urban are successful in access to debt financing compared those located in rural areas. Physical closeness between lenders and borrowers produce an improved form of environmental scrutinize that aid SMEs to access credit from lenders. Consequently, there is a positive relationship between firm’s location and access to debt financing by small businesses (Kira & He, 2012; Meressa, 2020).

There is some form of entrepreneurial continuum between voluntary and forced entrance conditions. Voluntary entrants have better entrepreneurial orientation than their forced entrant counterparts. Therefore, the degree of voluntary entrance can serve as proxy for the degree of entrepreneur ability. MSE owner who voluntarily started business has a relatively good deal of entrepreneurship behavior and relatively higher preference for external financing schemes (Gebru, 2009; Mersha & Ayenew, 2017). With regard to education, the higher the level of education of an entrepreneur, the easier it is to process information and adapt to the changing business world**.** MSSEs Managers who have better educational level can prepare business plan and financial statement which are very important predictors of access to finance from financial institutions (Amene, 2017). Small business owner-managers who had degrees generally have sustained advantages and provide an entrepreneur with a greater mental ability to be an innovator, make a decision that would bring successful outcomes, and impact positively because he is able to satisfy the demands of a changing job environment (Eniola, 2018). Therfore, level of education is a major factor that affects MSSEs’ access to credit from formal financial institutions. This probably is either because a higher education means that entrepreneurs are more articulate and more likely therefore to persuade the formal financial institutions that they have a viable proposition or because financial institutions value entrepreneurs with higher education (Kebede et al., 2014). With regard to location, geographic proximity to banks and customers has a relationship on a firm’s use of credit for the fact that banks that are geographically closer to their customer firms are better able to use soft qualitative information about their customers’ credit quality.Small eneterprises that are located in the towns and cities are more likely to be successful in their credit application compared to enterprises located in rural areas (Fatoki & Asah, 2011).

**Fig. 1:**
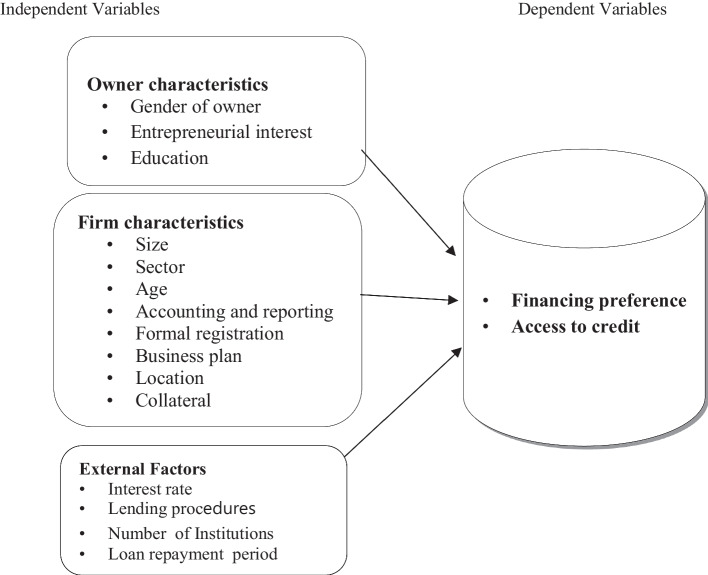
Conceptual framework

Moreover, business experience has its own effect on financing decision. Micro- and small-scale enterprises who do not have adequate experience follow the Pecking Order Theory when choose their capital sources (Al-Afifi, 2019). New enterprises are more likely to prefer low cost and less formal financing such as internal or bootstrap finances like grants, gift, sell of properties and hire purchase. However, as the enterprise gets established or matures, its capacity to seek formal financing increases, thereby becoming more likely to prefer or being in a higher category of formal financing (Awlachew & Motumma, 2017). Business experience can affect MSSEs’ access to finance in the sense that small business that have been in existence for a short period of time may find it difficult to access finance because they have not been tried and tested compared to their counterparts that have been in existence for longer (Zarook et al., 2013). So, young and small firms appear to face more serious financial constraints relative to those that are larger and more established (World Bank Group, 2015). That is why younger enterprises report higher financing obstacles even after controlling for other firm characteristics (Beck, 2007). Being in a business for many years suggests that the firm is competitive in general and more transparent so that the information required by lenders to evaluate and process applications is readily available. Moreover, new firms are not likely to meet the collateral requirements of the banks since they have not accumulated sufficient assets (Gamage, 2013). Not only the above variables, business plan is also determining factor of access to credit. Almost any investor today will require a firm to present a business plan to access credit. Therefore, business planning is significantly associated with debt access. Most firms cannot access loans because of the lack of realistic and workable business plans. Theoretically, a written business plan positively affects the capacity of small business to obtain loans. (Abdesamed & Abdesamed, 2014). Good business plan is perceived as one of the most essential documents to be prepared by small business since lack of adequate information leads to information asymmetry and credit rationing. Therefore, there is positive relationship between business information and access to debt finance by small business (Amene, 2017; Fatoki & Asah, 2011) (Fig. 1).

## Research methodology

### Research design and approach

The research design was correlational explanatory research design based on a deterministic philosophy in which causes probably determine outcome followed by quantitative approach. Deterministic philosophy believe that, with appropriate measurement tools, researchers can objectively uncover absolute, undeniable truths about cause and effect relationships (Bhattacherjee, 2012; Saunders et al., 2007). Moreover, quantitative research is an approach for testing objective theories by examining the relationship among variables that can be measured, typically on instruments, so that numbered data can be analyzed using statistical procedures (Creswell, Research design: qualitative, quantitative, & mixed methods approaches, 2014).

### Data collection instrument

The study used primary data collected from selected micro- and small-scale enterprises in Benishangul Gumuz Regional State of Ethiopia since 2019. The main instrument for data collection in this research was structured questionnaire. The questionnaire was prepared in English language. Reliablity and validity of the instrument was also checked. In this regard, it is evidenced in literature that reliability can be checked using test–retest measurements of the same construct administered to the same sample at two different points in time. Besides, validity can be assessed based on correlational coefficient of pilot test data in quantitative research (Bhattacherjee, 2012). In this study, the survey instrument was first reviewed by lecturers of accounting and finance department in Assosa University for validity and then pre-tested to evaluate its suitability on 30 piloted entrepreneurs. Thereafter, a test–retest method was used to examine the reliability of the instrument and the instrument was administered twice to the same group of subjects at an interval of one month and gave a correlation coefficient of 0.724 that indicates high reliability of the instrument for the fact that coefficient of 0.5 and above is deemed reliable (Kothari, 2004).

### Population, sample size and sampling technique

There were 1140 micro- and small-scale enterprises according to the data obtained from Benishangul Gumuz Regional State of MSSEs agency during 2019. The target population of the study was, therefore, all micro- and small business enterprises in the study area. Moreover, geographical and sectoral population distribution is given in Table 1.

**Table 1:** Population distribution

Sector	Geographical location								Sub-total
	Assosa	Bambasi	Homosha	Menge	Sherkole	Kurmuk	Oda	Mao-komo	
Manufacturing	49	6	5	1	3	0	0	1	65
Construction	277	17	10	32	11	1	4	22	374
Service	173	40	6	8	0	2	13	13	255
Agriculture	136	31	18	46	8	13	19	4	275
Trade	127	21	4	4	2	7	5	1	171
Total	762	115	43	91	24	23	41	41	1140
Percentage	67%	10%	4%	8%	2%	2%	3.5%	3.5%	100%

Indeed, sample size was determined based on the whole zonal active enterprises using a simplified formula developed by Yamane (1967):

$$\mathrm{Sample size}=\left(\frac{\mathrm{population size}}{1+\mathrm{population size}\left(\mathrm{level of }{\mathrm{precision}}^{2}\right)}\right),$$

$$\mathrm{Sample size}=\left(\frac{1140}{1+1140\left( {0.05}^{2}\right)}\right)=296,\mathrm{ where level of precision}= 5\mathrm{\%}$$

Therefore, representative of 296 enterprises were used from the target enterprises. With regard to sampling technique of the survey, the study used a combination of cluster, stratified and purposive sampling methods. First, four clusters (Assosa Wereda, Bambasi Wereda, Menge Wereda, and Homosha Wereda) were selected purposely due to their relative higher enterprises’ density. Thereafter, the enterprises were stratified in to manufacturing sector, construction sector, service sector, agricultural sector and trade to create sectoral homogeneity in each. Finally, proportional representative enterprises were selected through snowballing to come up with a total of 296 respondents as depicted in Table 2.

**Table 2:** Proportional distribution of representatives

Sector	Geographical location								Sub-total	
	Assosa		Bambasi		Homosha		Menge			
	Target	Sample	Target	Sample	Target	Sample	Target	Sample	Target	Sample
Manufacturing	49	14	6	2	5	2	1	0	61	18
Construction	277	81	17	5	10	3	32	9	336	98
Service	173	51	40	12	6	2	8	2	227	67
Agriculture	136	40	31	9	18	5	46	14	231	68
Trade	127	37	21	6	4	1	4	1	156	45
Total	*762*	*223*	*115*	*34*	*43*	*13*	*91*	*26*	*1011*	*296*

### Variable description and model specification

The choice of dependent and independent variables with their measurement is a matter of no choice while specifying an empirical model.

In line with this, dependent variables of this study were financing preference and access to credit by micro- and small-scale enterprises. In addition, independent variables were combination of owner’s characteristics, firm-related characteristics and creditor-related variables which are described in Table 3.

**Table 3:** Nature and measurement of variables

Variables	Nature	Measurement	Previous studies that used the same measurement
Financing preference	Categorical	1 if internal source preference and 0 debt	(Gebru, 2009; Kuruppu & Azeez, 2016)
Access to credit	Categorical	1 if enterprises have access to finance and 0 otherwise	(Kebede et al., 2014; Nguyen et al., 2015; Osebo, 2014; Zelalem & Wubante, 2019)
Collateral	Categorical	1 if there is collateral and 0 otherwise	(Kira, 2013; Mersha & Ayenew, 2017; Osano & Languitone, 2016; Osebo, 2014)
Firm size	Continuous	Number of employees	(Kira, 2013; Mersha & Ayenew, 2017; Nguyen et al., 2015; Osebo, 2014; Zarook et al., 2013)
Location	Categorical	1 = Assosa, 2 = Bambasi, 3 = Menge, 4 = Homosha	(Fatoki & Asah, 2011; Kira, 2013)
Business experience	Continuous	Number of years in business	(Abdesamed & Abdesamed, 2014; Kira, 2013; Mersha & Ayenew, 2017)
Gender of owner	Categorical	1 if male owned and 0 other wise	(Kebede et al., 2014; Nguyen et al., 2015; Osebo, 2014)
Enterprise’s sector	Categorical	1 = Manufacturing, 2 = Trade, 3 = Construction, 4 = Service and 5 = Agriculture	(Kebede et al., 2014; Kira, 2013; Mersha & Ayenew, 2017; Nguyen et al., 2015; Osebo, 2014; Zarook et al., 2013)
Interest	Categorical	1 if owners join by choice and 0 otherwise	(Gebru, 2009; Mersha & Ayenew, 2017)
Owner’s education	Categorical	4 = No formal education, 3 = elementary, 2 = secondary, 1 = vocational and university	(Abdesamed & Abdesamed, 2014; Kebede et al., 2014; Kira, 2013; Nguyen et al., 2015; Osebo, 2014)
Financial reporting	Categorical	1 if there is record keeping and 0 otherwise	(Amene, 2017; Kira, 2013; Mersha & Ayenew, 2017)
Interest rate	Categorical	1 if the entrepreneurs perceive that the cost of borrowing is low, otherwise 0	(Kebede et al., 2014)
Lending procedures	Categorical	1 if easy procedure, otherwise 0	(Kebede et al., 2014; Osebo, 2014)
Business plan	Categorical	1 if there is business plan and 0 otherwise	(Abdesamed & Abdesamed, 2014; Amene, 2017; Fatoki & Asah, 2011; Zelalem & Wubante, 2019)
Repayment period	Categorical	1 if long repayment and 0 if low duration	(Amene, 2017; Kebede et al., 2014; Osebo, 2014)

Coming to the model specification, logistic regression model was used to examine the relationship between independent variables and dependent variables (financing preference and access to credit of MSSEs). The basis for selecting the binary logistic regression model was the nature of both the dependent variables which are financing preference and access to credit. The first dependent variable, financing preference, was rated and reported by the respondents with a discrete scale of 1 and 0, where 1 is financing preference from internal sources and 0 for financing preference from external sources through credit in the first place. In the second stage, only entrepreneurs with external financing preference were considered and access to credit was rated and reported by the respondents with a discrete scale of 1 and 0, where 1 is access to credit with satisfied demand in the credit market and 0 otherwise. The baseline regression model for this study could therefore be econometrically specified based on normal cumulative distribution function model following Gujarati (2004) as:

1 $${\text{Pr}}\left( {y = {1}|x} \right) = F\left( {{\text{B1 + }}B_{{\text{n}}} {\text{xi}}} \right),$$

where *y* is a binary indicator equal to 1 if entrepreneurs reported internal source of financing and 0 otherwise in the first model with objective of investigating financing preference determinants. While addressing the second objective which is examining access to credit determinants, *y* is a binary indicator equal to 1 if entrepreneurs with external source of financing preference reported satisfaction in credit market demand and 0 otherwise. *F* is the normal cumulative distribution function; B1 and *B*_n_ are parameters to be estimated; and xi is a vector of explanatory variables that potentially may affect entrepreneurs’ financing preference and access to credit.

The main interest of the above equation is to investigate entrepreneurs’ financing preference determinants in the first place and to examine access to credit determinants in the second phase based on data collected from entrepreneurs with external source of financing preference. However, entrepreneurs with external source of financing preference are not randomly selected from the population and this may result in second-stage regression suffering from selection bias (Cameron & Trivedi, 2005). In addition, there is a potential reverse-causal relationship between enterprise size and access to credit in the second model. To justify this, on the one side, large enterprises are likely to build more assets and these assets could be deployed as collateral to enhance the probability of access to external financing from financial institutions in the credit market. In this regard, compared to enterprises with large size, enterprises with small size are less likely to access credit and accessing credit to micro business will be more difficult which may be out of reach compared to large and smaller firms (Awlachew & Motumma, 2017; Kebede et al., 2014). On the other side, access to external finance from credit market could improve enterprises’ prospects of expansion, increased profitability, and better capital base building that boosts firm size. In this regard, enterprises which have access to external finance grew better than those which have shortage of credit (Afande, 2015; Leza et al., 2016; Melesse, 2019). Because of this feedback loop, standard estimation techniques that take size of enterprises as exogenous, could give us inconsistent estimates. Therefore, as robustness check, the following two-step IV-probit model is estimated following (Melesse, 2019; Wooldridge, 2002):

2 $${\text{Pr}}\left( {y = {1}|x,z} \right) = F(x * d + {\text{IMR}}\left( {{\text{zw}}} \right) * \lambda ),$$

where the inverse Mills ratio is the ratio of the probability density function to the complementary cumulative distribution function of a distribution. The ratio is calculated from the first step by dividing the probability density function by the corresponding cumulative distribution function evaluated at the index zw. In the above equation, *z* and *x* are vectors of predictors. In the same fashion, *d*, *w*, and *λ* are parameters to be estimated. In this regard, testing the null hypothesis that *λ* = 0 is equivalent to asserting that enterprise size is independent of access to external financing.

### Method of data analysis

Data analysis is a significant methodological component while conducting empirical study. The literature documented that guidelines for conducting data analyses in quantitative studies are common, but often underemphasize on proper handling of missing data, proper level of measurement of variables and model checking to ensure statistical inferences are valid (Abulela & Harwell, 2020). In this regard, variables of the study were measured properly by referring empirical studies from the existing literature of small business financing and the collected data were screened and treated for errors and missing values. The study used both descriptive and inferential statistics to draw conclusions. For statistical treatment, statistical software for data science (STATA version 13) was used. Accordingly, the descriptive statistics was discussed first using tables and figures. The instrument’s reliability was also checked. In addition, Hosmer and Lemeshow test was used to determine the acceptability of the model in the logistic model. Moreover, regression analysis was carried out using marginal effect of probit output after checking existence of multicollinearity problem using Cramer's V. Finally, two robustness checks namely reversal causality and sample selection bias were performed on the baseline results.

## Empirical results and discussion

### Status of MSEs in Ethiopia and the position of enterprises in Benishangul Gumuz Region

To date, micro- and small-enterprise development has been top on the agenda of the government of Ethiopia as an instrument to achieve the triple objectives of poverty reduction, employment expansion and economic growth (Melesse, 2019). In this regard, on average a total of 169, 797 new micro- and small-scale enterprises were established during the last eight years. Nationally, the number of enterprises established in 2012, 2013, 2014 and 2015 were 206,352, 77,415,200,319 and 271,579, respectively. In the same vein, 157,768, 144,107, 110,253 and 190,587 enterprises were established during 2016, 2017, 2018 and 2019, respectively. The maximum enterprises establishment was registered since 2015 (insert Table 7 in the appendix). With regard to employment, on average, micro- and small-scale enterprises have created job for 1, 403,011 within 8 consecutive years. To look at the annual trend, the number of employment created in 2012, 2013, 2014 and 2015 was 806,322, 1,223,679, 2,497,181 and 2,788,667, respectively. In the same vein, 11,665,517, 1,172,678, 187,945, 882,098 job was created during 2016, 2017, 2018 and 2019, respectively, at country level. Accordingly, the maximum job was created since 2015 (insert Table 8 in the appendix).

**Fig. 2:**
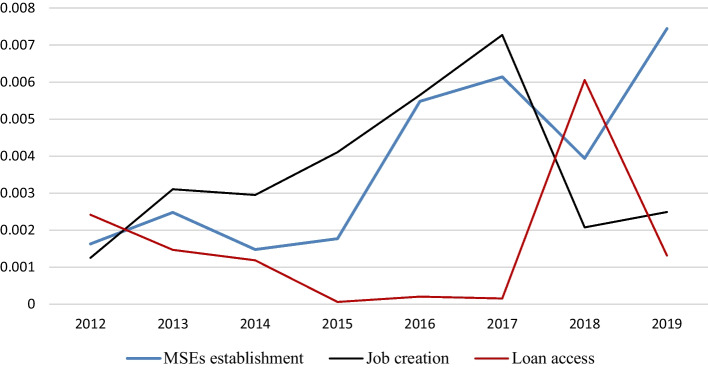
Position of MSEs in BGRS relative to enterprise at national level

The status of loan created to the sector was also assessed. In this regard, a total of 141,357 million credit was created within 8 years on average. Accordingly, the amount of loan created in 2012, 2013, 2014 and 2015 were 108,813.7, 2725.1, 5063.9 and 6541.88, respectively. In the same vein, 5366.6, 7075.8, 8633.7 and 7311.8 loan was created during 2016, 2017, 2018 and 2019, respectively. The biggest loan was created in 2012 according to the report of NBE. In every year, with regard to the parameters discussed above, the share of Benishangul Gumuz Region is among the lowest shares relative to other regions (Insert Table 9 in the appendix) and the position of MSEs in the region is depicted in Fig. 2.

With regard to financing preference, majority of the enterprises, almost 54.73% of the enterprises prefer to finance their businesses by external source of fund through credit as depicted in Table 4. On the other side, 45.27% prefer to raise fund internally from their own source. In this study, five operational sectors were incorporated including enterprises engaged in trade, service, agricultural, construction and manufacturing sectors. Accordingly, the evidence indicates that 6.08% are engaged on agricultural sector, 15.2% are engaged on trade sector, 33.11% are engaged on construction, 22.64% are engaged on service sector, and the remaining 22.9% are engaged on Manufacturing sector. The survey revealed that majority of the respondents with 70.94% had operated their businesses for a period of fewer than three years followed by 26.69% with business experience that ranges between 4 and 5 years while those who had been in operation for more than five years shared the least percentage which is 2.37%. Therefore, majority of the enterprises are at their start up stage.

**Table 4:** Descriptive statistics

Items		Frequency	Percent
Financing preference	External source through credit	162	54.73
	Internal source	134	45.27
	Total	296	100.00
Sectoral engagement of enterprises	Agriculture	18	6.08
	Trade	45	15.20
	Construction	98	33.11
	Service	67	22.64
	Manufacturing	68	22.97
	Total	296	100.00
Business experience	1–3 years	210	70.94
	4–5 years	79	26.69
	Above 5 years	7	2.37
	Total	296	100.00
Gender	Female	120	40.54
	Male	176	59.46
	Total	296	100.00
Entrepreneurial interest	Not motivated	170	57.43
	Motivated	126	42.57
	Total	296	100.00
Access to land	Private owned	164	55.41
	Rented	47	15.88
	Government	85	28.72
	Total	296	100.00

Source: author’s computation based on enterprise survey (2019)

The results of the questionnaire indicated that 40.54%) were female and the remaining 59.46% were male. Among the enterprises, with regard to motivation of owners, almost 57.43% of the owners did not join to the sector by their choice and the rest 42.57% of the owners joined to their business with interest. Among the enterprises, majority of the respondents in the survey (55.41%) use their own land for operation, 15.88% operated their businesses on rented houses while the remaining percentage (28.72%) operate their business using premises from the government. The survey revealed that 62.96% of the respondents replied that their request for loan was accepted by lenders whereas 37.04% of the respondents reported rejection of their request. The study revealed that among the enterprises that prefer external debt as means of financing, 5.56% are engaged on agricultural sector, 17.28% are engaged on trade sector, 29.63% are engaged on construction, 23.46% are engaged on service sector, and the remaining 24.04% are engaged on Manufacturing sector. Therefore, relatively, more of the enterprises engaged in construction sector Prefer external source of financing. On the one side, according to the survey, 40.74% of owners of micro and small enterprises responded that they prepare business plan which could assist the operation of their businesses. On the other hand, 59.26% of the owners answered that they do not have business plan for their business and described that they faced number of problems one of which being lack of access to loan because business plan is a proposal that describes a business opportunity to financing. Among the enterprises that need to finance their operation from external sources through credit, 75.31% were operated in Assosa Wereda, 12.35% Bambasi Wereda, 8.02% in Menge and 4.32% were operating in Oda.

### Econometric results and discussion

In this section regression analysis is carried out using marginal effect of probit output. In this study, the econometric analysis section consists of two-step analysis. The first step is used to analyze financing preference of entrepreneurs to test pecking order theory of capital structure using logistic regression model. On the second step, only enterprises that prefer external source of financing through credit are considered. Prior to running logistic regression, however, explanatory variables were checked for the existence of multicollinearity problem using Cramer's V for the fact that there are many discrete variables. Cramer's V is a measure for the strength of an association between two discrete variables in tables bigger than 2 by 2 tabulation. Cramer's V varies between 0 and 1 without any negative values (Akoglu, 2018). The result revealed that there is no collinearity problem within the data as shown in the Cramer’s V contingency correlation test result (Insert Table 10 in the appendix). In addition, the Hosmer and Lemeshow test was used to look at goodness of fit in the logistic model suggesting that the model is quite a good fit with Prob > chi^2^ = 0.2369.

As indicated in Table 5, econometric results from probit estimation on financing preference of entrepreneurs’ show that business experience, collateral, gender, motivation owner’s education and access to land premise are significant in explaining financing preference of entrepreneurs. The other variables, however, failed to show significant influence.

**Table 5:** Determinants of financing preference (marginal effects from probit estimation)

Variable	d*y*/d*x*	Std. err.	*Z*	*P* >|*z*|
Business experience	0.2206916	0.0665383	3.32	0.001
Enterprise size	0.0110633	0.0101499	1.09	0.276
Sectoral engagement	Manufacturing sector as reference			
Trade sector	− 0.5764228	0.3886996	− 1.48	0.138
Construction sector	0.0475746	0.3547028	0.13	0.893
Service sector	− 0.2723631	0.3698208	− 0.74	0.461
Agriculture sector	− 0.1058209	0.3681426	− 0.29	0.774
Collateral	0.8333717	0.1682877	4.95	0.000
Gender	0.6296639	0.1703395	3.70	0.000
Entrepreneurial interest	0.2805849	0.1687058	1.66	0.096
Education	Vocational and university as reference			
Secondary	− 0.2504659	0.1804218	− 1.39	0.165
Elementary	− 0.4780596	0.2340156	− 2.04	0.041
No formal education	− 0.4386958	0.6080814	− 0.72	0.471
Land	Own land premise as reference			
Government	− 0.4834399	0.2428885	− 1.99	0.047
Rental	− 0.2534156	0.1843556	− 1.37	0.169

Ceteris paribus, as described in Table 5 above, gender of micro- and small-scale enterprises’ owners was found to have positive relation with financing decision of MSSEs in light with pecking order theory and statistically significant at 1 percent. The marginal effect of this variable shows that the probability of choosing internal financing for male owned MSSEs increases by 62.96 percent as compared to female owned MSSEs. Therefore, it is evident that male owned MSSEs more likely prefer internal financial sources as compared to female owned MSSEs. The result of this study contradicts with Kuruppu and Azeez (2016) which found internal financing source preference of female entrepreneurs. Contrary to this, it is documented that women MSME owners are more conformances to POT than men. This might be due to many factors such as male culture in the works, preference for men in the high level of the works (Al-Afifi, 2019).

With regard to entrepreneurial interest of micro- and small-scale enterprises’ owners, it was found to have positive relation with financing decision of MSSEs following pecking order theory and statistically significant at 10 percent. The marginal effect of this variable shows that the probability of choosing internal financing for motivated MSSEs’ owners increases by 28. 05 percent as compared to their counterparts. Therefore, it is found that motivated entrepreneurs more likely prefer internal financial sources supporting pecking order hypothesis. This result contradicts with the finding of a study made by Gebru (2009) in MSSEs of Tigray Regional State which revealed voluntary entrants have better entrepreneurial orientation than their forced entrant counterparts for the fact that the MSE owners who voluntarily started business have a relatively good deal of entrepreneurship behavior and relatively higher preference for external financing scheme.

In addition, collateral of micro- and small-scale enterprises was found to have positive relation with financing decision of MSSEs owners and statistically significant at 1 percent in light of pecking order hypothesis. The marginal effect of this variable shows that the probability of choosing internal financing for MSSEs’ owners who have collateral increase by 83.33 percent as compared to their counterparts in light of pecking order theory of financing decision. Business experience, measured by year of establishment also revealed a positive effect on financing decision at 1 percent significant level statistically in favor of pecking order theory of financing decision. The marginal effect implies that, ceteris paribus, the probability of choosing internal financial source increases by 22.06 percent as business experience increases by a year. This result contradicts with Awlachew and Motumma (2017) which found internal financing source preference of newly established firms. Moreover, access to land premise of micro- and small-scale enterprises’ owners was found to have negative relation with financing decision of MSSEs’ owners and statistically significant at 5 percent. The marginal effect of this variable shows that the probability of choosing internal financing for MSSEs’ owners with governmental land premise decreases by 48.34 percent as compared to those who own land premise. Finally, owners/ managers of micro- and small-scale enterprises’ educational status was found to have negative relation with financing decision and statistically significant at 5 percent. The marginal effect of this variable shows that the probability of choosing internal financing for MSSEs’ owners with educational status of elementary level decreases by 47.8 percent as compared to those who join vocational centers and universities. In the literature, however, it is documented that the financial illiterates of micro- and small-scale owners and who with less educated are more comfortable with pecking order theory (Al-Afifi, 2019).

In the following logistic regression result, only those enterprises with external financing preference were considered and access to credit refers to demand satisfaction of entrepreneurs in the credit market. In this regard, satisfied demand of credit was indicated by ‘1’ and ‘unsatisfied demand of entrepreneurs indicated by ‘0’.

As indicated in the probit estimation output, in Table 6, business experience has a positive effect on access to credit at 1 percent significant level statistically. The marginal effect implies that, ceteris paribus, the probability of accessing credit increases by 14.4 percent as business experience increases by a year. The finding of this study is consistent with Beck (2007); Zarook et al., (2013) and Gamage (2013) suggesting that being in a business for many years indicates that the firm is competitive in general and more transparent so that any information required by lenders to evaluate and process applications is readily available relatively. Enterprise size has also a positive and statistically significant effect on MSSEs’ access to credit at 5% level of significance. The marginal effect shows that the probability of accessing credit increases by 11.53 percent for MSSEs as employee number increases by a unit. This result is consistent with Fufa (2016) implying that lenders base their lending decisions on the size of firms and credit approval is easy for firms as enterprise size increases. The probit estimation results indicate that enterprises’ location has significant impact in accessing external credit at 10 percent significance level statistically. The marginal effect shows that the probability of accessing credit increases by 19.17 percent for those MSSEs that are locate in Bambasi Wereda than those operated in Assosa Wereda.

**Table 6:** Determinants of access to credit (marginal effects from probit estimation)

Variable	d*y*/d*x*	Std. err.	*z*	*P* >|*z*|
Business experience	0.1442775	0.0242488	5.95	0.000
Enterprise size	0.1153693	0.0581971	1.98	0.047
Sectoral engagement	Manufacturing sector as a reference			
Trade sector	− 0.0692217	0.1329692	− 0.52	0.603
Construction sector	− 0.0616193	0.1232409	− 0.50	0.617
Service sector	0.0460313	0.1281724	0.36	0.719
Agriculture sector	− 0.0406567	0.1196771	− 0.34	0.734
Collateral	0.1813233	0.0539846	3.36	0.001
Gender	0.0081331	0.0550723	0.15	0.883
Interest rate	− 0.1289983	0.0478643	− 2.70	0.007
Loan repayment period	− 0.118962	0.0715457	− 1.66	0.096
Financial reporting	0.2245718	0.0568789	3.95	0.000
Lending procedures	0.0535897	0.0660112	0.81	0.417
Formal registration	0.1026668	0.0485263	2.12	0.034
Business plan	0.1790045	0.051102	3.50	0.000
Location	Assosa Wereda as a reference			
Bambasi	0.1365449	0.070228	1.94	0.052
Menge	0.1184196	0.0746654	1.59	0.113
Homosha	0.0465122	0.1135408	0.41	0.682
Number of institutions	0.0060085	0.0646789	0.09	0.926
Education	Vocational and university as reference			
Secondary	− 0.0691527	0.0588094	− 1.18	0.240
Elementary	− 0.0573439	0.0667854	− 0.86	0.391
No formal education	0.0566267	0.1607418	0.35	0.725

The output of probit estimation also revealed that collateral has a positive and statistically significant effect on MSSEs’ access to credit at 1% level of significance level statistically. The marginal effect shows that the probability of accessing credit increases by 18.13 percent for those MSSEs that have collateral than their counter parts. This result is consistent with finding of studies made by Kebede et al., (2014), Osebo (2014), Mole and Namusonge (2016) which found high demand of collateral by financial institution to make credit for their security. In addition, interest rate has a negative and statistically significant association with MSSEs’ access to credit at 1 percent significance level statistically. The marginal effect shows that the probability of accessing credit decreases by 12.89 percent for those MSSEs’ owners who believe that interest rate of loan is higher than their counter parts. Loan repayment period also has a negative and statistically significant association with MSSEs’ access to credit at 10% significance level statistically. The marginal effect shows that the probability of accessing credit decreases by 11.89 percent for those MSSEs’ owners who believe that loan repayment period short than their counter parts who perceive that loan repayment period is long enough.

With regard to financial reporting, the output of probit estimation revealed that financial reporting has a positive and statistically significant effect on MSSEs access to credit at 1% level of significance statistically. The marginal effect shows that the probability of accessing credit increases by 22.45 percent for those MSSEs that prepare financial reporting than their counter parts. This result is consistent with Fanta (2015) which found producing financial statements enhances the chance to secure bank credit. Moreover, business plan affected access to credit statistically at 1 percent significance level. The marginal effect indicates that the probability of MSSEs access to credit with business plan increases by 17.9% compared to MSSEs that do not have business plan at 1 percent level of significance statistically. Theoretically, a written business plan positively affects the capacity of small business to obtain loans (Abdesamed & Abdesamed, 2014). This result is consistent with Fatoki and Asah (2011) that found positive relationship between preparation of business information and access to debt finance by small business. Finally, formal registration of enterprises has a positive and significant influence on MSSEs’ access to finance at 5 percent significance level statistically. The marginal effect shows that the probability of accessing credit increases by 10.26 percent for those MSSEs’ owners who have formal registration than their counter parts.

### Robustness check of results from standard probit estimation

To date, robustness tests have become an integral part of research methodology. Robustness tests are all about assumptions. It is necessary for valid causal inference, in that the coefficients of the critical core variables should be insensitive to adding or dropping variables (Lu & White, 2014). In this regard, two robustness checks namely reversal causality and sample selection bias were performed on the baseline results. With regard to reversal causality, the paper examines the possibility of enterprise size being endogenous to access to credit using two-step IV-probit. In this regard, enterprise size was predicted as a function of instruments including market linkage, entrepreneurs’ motivation and work experience and other covariates namely access to credit, business experience proxied by age of enterprise, sectoral engagement, gender of owner/manager, educational status of owner/manager and location of enterprise using a probit specification to get the index zw in the first step which was used to calculate inverse mills ratio. Following this, Wald test of exogeneity was used to check the causality problem. However, the test provided insignificant result (Insert Table 11 in the appendix). To put it in other way, the Chi-square is 0.58 with *p* < 0.4459) and the estimated coefficient of athrho is − 0.1006607 with *p* < 0.446. Therefore, the insignificant result of Wald test of exogeneity (athrho = 0) indicates that size of enterprise is not truly endogenous and estimation of a standard probit regression produced efficient and unbiased estimators. Finally, the marginal effect estimates from the two-stage instrumental variable probit model by including the inverse mills ratio as additional regressors were carried out though there is no endogeneity of reversal causality to look at the difference (insert Table 12 in the appendix).

Lastly, enterprises with external source financing preference through credit are not randomly selected from the population. In this regard, the result in second-stage regression may suffer from selection bias (Cameron & Trivedi, 2005). Accordingly, the coefficients discussed above may not be applicable to all enterprises if sample-induced endogeneity due to selectivity exists. To check sample-induced endogeneity, therefore, Heckman two-stage process was used. To do Heckman’s two steps, an intuitive way is to estimate the selection equation first to predict inverse mills ratio and include it as additional regressors in the outcome equation then after. In the first stage, therefore, financing preference was estimated on business experience, sectoral engagement, size of enterprise, collateral, gender, entrepreneurial interest, educational status and access to land premise using probit model. Followed by, zw (the sum of each variable evaluated at its mean value multiplied by its probit estimate) was predicted based on the selection output. Then after, standard normal probability density function and cumulative distribution function were calculated to construct inverse mills ratio. In the second stage, inverse mills ratio was included as additional regressors in the outcome equation to examine its significance. However, inverse mills ratio is not significant (reported in Table 13 in appendix**)** though negatively signed. Therefore, sample-induced endogeneity is not a problem here suggesting that the error terms in the selection equation and outcome equation are not correlated significantly and estimate discussed above is free of selection bias. While performing the test, entrepreneurial interest and access to land premise were included only in the selection equation as exclusion restriction for a robust identification in the outcome equation.

## Conclusion, implication and direction for future research

The purpose of this study was to examine factors that determine micro- and small-scale enterprises’ financing preference in line with pecking order theory and access to credit in Benishangul-Gumuz Regional State of Ethiopia using logistic regression via two-stage analysis. The first step was used to analyze financing preference of entrepreneurs and its determinants to test whether the firms followed pecking order hypothesis in their financing decision using data collected from 296 enterprises. The evidence revealed that almost 54.73% (162) of the enterprises prefer to finance their businesses by external source of fund through credit. On the other side, 45.27% (134) prefer to raise fund internally from their own source. Therefore, majority of the entrepreneurs did not follow a financing decision suggested by pecking order hypothesis. The results of logistic regression analysis revealed that business experience, collateral, gender, motivation and enterprises’ sectoral engagement affect financing preference of enterprises. In the second step, only enterprises that need to raise capital through credit (162 enterprises) were considered to investigate the determinants of access to credit. Accordingly, the survey revealed that 62.96% (102) of the respondents replied that their request for loan was accepted by lenders, whereas 37.04% (60) of the respondents reported rejection of their request. The logistic regression result revealed that business experience, size, sectoral engagement, collateral, interest rate, loan repayment period, financial reporting, preparation of business plan, location and educational status of entrepreneurs affect access to credit of enterprises. In the literature, it is evidenced that research is important for three reasons. First, it is undertaken to contribute to existing information about issues by providing additional results to confirm or disconfirm results of prior studies and add value to existing knowledge. Second, it is undertaken to suggest improvements for managerial practice. Third, it provides information to policy makers (Creswell, 2012). In this regard, the following implications are provided based on the finding.

### Managerial implication

The managerial implication of the study is that it might help the entrepreneurs in addressing the factors affecting financing preference and access to credit to take actions towards developing their performance by choosing best financial decision. Therefore, owners of small businesses should share business experience, improve entrepreneurial interest, prepare business plan for the fact that these variables are powerful in explaining outcomes of the study. The owners are also suggested to establish lasting and trusting relationship with creditors to mitigate asymmetric information in order to obtain favorable terms of credit by producing quality financial report as long as asymmetric information restrain the enterprises from accessing external loans. Furthermore, the owner-managers of the enterprises must make a conscious effort to improve their financial knowledge by attending workshops, conferences and short courses on financial reporting and business plan preparation in which the events could be organized through enterprise–university collaborations.

### Theory/literature implication

Despite a large number of studies have been documented on financing decision, surprisingly there are insignificant number of studies carried out to examine financing preference determinants of small business particularly for enterprises of developing countries in which Ethiopia is inclusive. This paper, therefore, contributes to fill the gaps in the literature by empirically investigating the determinants of financing preference in line with pecking order theory and access to credit in Benishangul Gumuz Regional State of Ethiopia. Therefore, the current study contributes to the pecking order theory in the context of small business sector in one regional state of Ethiopia as developing economy.

### Policy implication

The results of this study are not only relevant to the owners of enterprises, but also to policy makers. The government of Ethiopia in its development strategy considered micro- and small business as engine of economic growth through employment creation though it is always recognized that access to finance is a major constraint of small businesses. Therefore, government needs to examine different options to finance and promote the enterprises. Government authorities are also suggested to assist in setting up credit guarantee schemes or providing funds for small businesses, which may reduce the asymmetric information between lenders and borrowers. Moreover, policymakers are suggested to adopt a user-friendly accounting and reporting system that will encourage micro and small businesses to be more transparent in financial dealings. This may improve the ability of financial institutions to assess creditworthiness of loan applicants and indirectly assist enterprises in getting better access to external finance which could boost their growth.

### Limitations of the study and direction for future research

There are many limitations associated with this paper although it has its own contribution to the literature of small business finance. This study examined micro- and small-scale enterprises’ financing preference and access to credit determinants regardless of their industry characteristics using structured questionnaire. However, a onetime structured questionnaire may not provide a detail understanding about the situation. Therefore, it is believed that future panel surveys and availability of other data with in-depth qualitative data may be important in order to have a comprehensive solution for the access to finance constraining factors. In addition, the results are representatives of micro- and small-scale enterprises in Benishangul Gumuz Regional State of Ethiopia and are not necessarily generalizable to other regions. Therefore, the result may be limited to the context of enterprises in Benishangul Gumuz and may not necessarily reflect financing decision behaviors and access to credit in other regions. Therefore, the study recommends that further research should be done on a larger sample of entrepreneurs all over the country. Another consideration that should be noted is that future research could look in more detail at the financing preference and access to credit determinants among small business entrepreneurs by conducting a comparative study of the Ethiopian situation with other developing countries. Lastly, the study was conducted before the abnormal situations of COVID-19. Therefore, the effect of COVID-19-related cases and lockdown measures on financing preference and access to credit can be used for future research agenda and comparative study can be conducted focusing on before and after the pandemic. This is for the fact that businesses have been exposed to various challenges during the global pandemic (Gregurec et al., 2021) and financing decision is inclusive.

## Data Availability

The data that support the findings of the study can be obtained from the author based on request.

## Appendix

See Tables 7, 8, 9, 10, 11, 12, 13.

**Table 7:** Number of micro- and small enterprises

Year	AA	Oromia	SNNRP	Amhara	Tigray	Dire	Harar	BGRS	Somalia	Gambela	Afar
2012	10,639	20,555	9819	99,750	64,000	333	265	336	519	109	27
	0.0516	0.0996	0.0476	0.4834	0.3101	0.0016	0.0013	0.0016	0.0025	0.0005	0.0001
2013	7571	16,897	7500	29,848	13,659	1482	118	192		83	65
	0.0978	0.2183	0.0969	0.3856	0.1764	0.0191	0.0015	0.0025		0.0011	0.0008
2014	7392	70,259	22,632	64,135	32,726	2017	240	296	222	309	91
	0.0369	0.3507	0.1130	0.3202	0.1634	0.0101	0.0012	0.0015	0.0011	0.0015	0.0005
2015	7291	140,858	19,521	65,480	33,570	2725	414	481	471	369	399
	0.0268	0.5187	0.0719	0.2411	0.1236	0.0100	0.0015	0.0018	0.0017	0.0014	0.0015
2016	8081	42,775	12,549	84,890	37,523	2124	372	1045	680	543	5
	0.0424	0.2244	0.0658	0.4454	0.1969	0.0111	0.0020	0.0055	0.0036	0.0028	0.000
2017	5540	49,373	20,474	38,692	39,600	1938	359	969	380	426	17
	0.0351	0.3129	0.1298	0.2452	0.2510	0.0123	0.0023	0.0061	0.0024	0.0027	0.0001
2018	9564	34,886	23,374	40,674	31,556	917	348	568	1600	547	73
	0.0664	0.2421	0.1622	0.2822	0.2190	0.0064	0.0024	0.0039	0.0111	0.0038	0.0005
2019	6730	33,047	10,021	32,123	23,256	1157	465	821	2053	580	–
	0.0610	0.2997	0.0909	0.2914	0.2109	0.0105	0.0042	0.0074	0.0186	0.0053	
Aver	7851	51,081	15,736	56,949	34,486	1586	322	588	740	370	84
	0.0462	0.3008	0.0927	0.3354	0.2031	0.0093	0.0019	0.0035	0.0044	0.0022	0.0005

Source: authors’ computation based on annual report of NBE from 2012–2019

**Table 8:** Total number of employment created by micro- and small enterprises

Year	AA	Oromia	SNNRP	Amhara	Tigray	Dire	Harar	BGRS	Somalia	Gambela	Afar
2012	99,899	267,474	127,112	174,971	82,680	45,248	3224	1010	3303	1401	–
	0.124	0.332	0.158	0.217	0.103	0.056	0.004	0.001	0.004	0.002	
2013	219,064	596,035	93,204	152,713	132,697	10,838	3728	3800	8710	1972	918
	0.179	0.487	0.076	0.125	0.108	0.009	0.003	0.003	0.007	0.002	0.001
2014	251,399	1,113,741	320,956	460,297	289,885	24,561	12,663	7373	11,009	3262	2035
	0.101	0.446	0.129	0.184	0.116	0.010	0.005	0.003	0.004	0.001	0.001
2015	277,587	1,531,028	190,945	525,391	178,886	26,073	6505	11,457	18,290	4185	18,320
	0.100	0.549	0.068	0.188	0.064	0.009	0.002	0.004	0.007	0.002	0.007
2016	189,866	530,989	161,001	582,508	140,518	25,125	8061	9413	12,533	3726	1777
	0.114	0.319	0.097	0.350	0.084	0.015	0.005	0.006	0.008	0.002	0.001
2017	80,966	474,592	202,675	187,514	164,157	23,201	8947	8530	16,905	3479	1712
	0.069	0.405	0.173	0.160	0.140	0.020	0.008	0.007	0.014	0.003	0.001
2018	6563	79,044	37,679	37,982	17,630	3380	572	390	2427	1112	1166
	0.035	0.421	0.200	0.202	0.094	0.018	0.003	0.002	0.013	0.006	0.006
2019	124,368	359,669	104,613	170,633	71,399	19,673	5257	2197	17,400	4574	2315
	0.141	0.408	0.119	0.193	0.081	0.022	0.006	0.002	0.020	0.005	0.003
Av	156,214	619,071.5	154,773.125	286,501.125	134,731.5	22,262.375	6119.625	5521.25	11,322.125	2963.875	3530.375
	0.1113	0.4412	0.1103	0.2042	0.0960	0.0159	0.0044	0.0039	0.0081	0.0021	0.0025

Source: authors’ computation based on annual report of NBE from 2012 to 2019

**Table 9:** Amount of credit created to micro- and small enterprises (in millions of birr)

Year	AA	Oromia	SNNRP	Amhara	Tigray	Dire	Harar	BGRS	Somalia	Gambela	Afar
2012	447,485.4	133,084.2	104,759.6	64,600.7	327,059.2	4029.8	6275.1	263	580.4	–	–
	0.4112	0.1223	0.0963	0.0594	0.3006	0.0037	0.0058	0.0002	0.0005		
2013	808.3	314.2	126.6	980	459.8	12.5	17	4	2.9	3.4	
	0.2966	0.1153	0.0465	0.3596	0.1687	0.0046	0.0062	0.0015	0.0011	0.0012	0.0000
2014	1567.4	755.6	338.7	1327.1	994.1	43.3	17.1	6	7.5	7.2	0
	0.3095	0.1492	0.0669	0.2621	0.1963	0.0086	0.0034	0.0012	0.0015	0.0014	0.0000
2015	2105.27	584.08	436.59	2071.07	1118.62	68.66	9.55	0.4	143.6	3.54	0.499
	0.3218	0.0893	0.0667	0.3166	0.1710	0.0105	0.0015	0.0001	0.0220	0.0005	0.0001
2016	1009.5	2046.4	957.1	938.5	240.7	59.8	18.2	1.1	85.6	4.6	4.9
	0.1881	0.3813	0.1783	0.1749	0.0449	0.0111	0.0034	0.0002	0.0160	0.0009	0.0009
2017	1641.3	1133.6	361.7	2750.3	882.8	160.3	29.2	1.1	114.9		0.7
	0.2320	0.1602	0.0511	0.3887	0.1248	0.0227	0.0041	0.0002	0.0162		0.0001
2018	1855	1005.6	1004.9	2479.1	1497.1	143.1	44.6	52.3	499.8	21.7	30.5
	0.2149	0.1165	0.1164	0.2871	0.1734	0.0166	0.0052	0.0061	0.0579	0.0025	0.0035
2019	1706.9	583	621.5	1318.1	2594	115.8	168.9	9.6	139.4	30.1	24.6
	0.2334	0.0797	0.0850	0.1803	0.3548	0.0158	0.0231	0.0013	0.0191	0.0041	0.0034
	57,272.4	17,438.3	13,575.8	9558.1	41,855.8	579.2	822.5	42.2	196.8	8.8	7.6
	0.40516	0.12336	0.09604	0.06762	0.29610	0.00410	0.00582	0.00030	0.00139	0.00006	0.00005

Source: authors’ computation based on annual report of NBE from 2012 to 2019

**Table 10:** Cramer’s *V* contingency correlation test results to check co-linearity among categorical variables

Variable	Sector	Collater	Interest	Repay	Gender	Report	Proced	Registr	Plan	Locat	Educati	Instituti
Sec	1											
Collat	0.158	1.000										
Inter	0.137	0.016	1.000									
Repay	0.162	0.222	0.139	1.000								
Gen	0.130	0.080	0.088	0.208	1.000							
Repor	0.166	0.044	0.140	0.286	0.043	1.000						
Proced	0.188	0.050	0.026	0.172	0.254	0.125	1.000					
Registra	0.137	0.112	0.103	0.173	0.044	0.289	0.091	1.000				
Plan	0.072	0.182	0.041	0.161	0.096	0.153	0.010	0.112	1.000			
Locat	0.174	0.188	0.102	0.210	0.132	0.185	0.145	0.083	0.100	1.000		
Educat	0.172	0.132	0.163	0.317	0.229	0.261	0.197	0.182	0.246	0.217	1.000	
Instit	0.317	0.078	0.128	0.177	0.057	0.072	0.140	0.002	0.136	0.288	0.110	1.000

**Table 11:** First stage IV-probit, enterprise size where small = 1 and 0 = micro

Variable	Coef.	*z*	*P* >|*z*|
Access to credit	0.7760535	0.67	0.008
Business experience	− 0.4029653	− 3.21	0.001
Sectoral engagement	Manufacturing sector as a reference		
Trade sector	1.075446	1.91	0.056
Construction sector	0.6824603	1.36	0.174
Service sector	0.8354956	1.61	0.107
Agriculture sector	0.3409372	0.67	0.501
Gender	0.0998092	0.42	0.676
Education	Vocational and university as reference		
Secondary	− 0.0978989	− 0.36	0.719
Elementary	0.2953994	0.91	0.364
No formal education	0.2434928	0.28	0.780
Location	Assosa Wereda as a reference		
Bambasi	0.0458861	0.12	0.902
Menge	− 0.5074518	− 1.20	0.232
Homosha	− 0.5857877	− 1.13	0.257
Constant	0.532604	0.99	0.321
/athrho	− 0.1006607	− 0.76	0.446
/lnsigma	− 2.051812	− 36.93	0.000
Rho	− 0.1003221		
Sigma	0.1285019		
Wald test of exogeneity (/athrho = 0): chi2(1) = 0.58 Prob > chi2 = 0.4459			

**Table 12:** Second stage probit: enterprise’s access to credit yes = 1 and no = 0

Variable	Coef.	*z*	*P* >|*z*|
Business Experience	0.7231269	2.22	0.026
Enterprise size	0.7541259	1.70	0.090
Sectoral engagement	Manufacturing sector as a reference		
Trade sector	0.3925442	0.33	0.743
Construction sector	− 0.3608476	− 0.40	0.688
Service sector	0.8627145	0.83	0.406
Agriculture sector	− 0.0308395	− 0.03	0.972
Collateral	0.1644788	0.17	0.867
Gender	− 0.9507408	− 1.08	0.282
Education	Vocational and university as reference		
Secondary	− 0.8487665	− 1.62	0.106
Elementary	− 0.1442227	− 0.27	0.789
No formal education	0.9305616	0.69	0.487
Interest rate	− 0.9658867	− 2.52	0.012
Loan repayment period	− 0.8612572	− 1.60	0.110
Financial report	1.588894	3.33	0.001
Lending procedure	0.3281761	0.69	0.491
Formal registration	0.5670824	1.46	0.144
Business plan	0.9532479	1.99	0.047
Location	Assosa Wereda as a reference		
Bambasi	1.047531	1.81	0.071
Menge	0.7714974	1.21	0.225
Homosha	0.2143832	0.26	0.798
Number of institutions	0.0998386	0.21	0.832
Inverse mills ratio	− 2.077474	− 1.27	0.203
Constant	0.9660338	0.28	0.780

**Table 13:** Heckman two-step procedures: coefficient estimates from selection bias model

Variable	First stage probit (Did the entrepreneurs prefer credit financing?)			Second stage Heckit (was the demand of entrepreneurs for accessing credit satisfied?)		
	Coef.	*z*	*P* >|*z*|	Coef.	*z*	*P* >|*z*|
Constant	− 1.39181	− 3.18	0.001	0.9660338	0.28	0.780
Business Experience	0.236284	3.62	0.000	0.7231269	2.22	0.026
Enterprise size	0.070358	0.39	0.698	0.7541259	1.70	0.090
Sectoral engagement	Manufacturing sector as a reference					
Trade sector	− 0.6156041	− 1.60	0.111	0.3925442	0.33	0.743
Construction sector	− 0.005312	− 0.02	0.988	− 0.3608476	− 0.40	0.688
Service sector	− 0.3226142	− 0.87	0.383	0.8627145	0.83	0.406
Agriculture sector	− 0.179787	− 0.49	0.624	− 0.0308395	− 0.03	0.972
Collateral	0.8113396	4.88	0.000	0.1644788	0.17	0.867
Gender	0.6440517	3.79	0.000	− 0.9507408	− 1.08	0.282
Entrepreneurial interest	0.3448571	2.10	0.036			
Education	Vocational and university as reference					
Secondary	0.2664668	1.48	0.138	− 0.8487665	− 1.62	0.106
Elementary	− 0.2235926	− 0.98	0.328	− 0.1442227	− 0.27	0.789
No formal education	− 0.2803545	− 0.48	0.630	0.9305616	0.69	0.487
Land	− 0.152858	− 1.67	0.095			
Interest rate				− 0.9658867	− 2.52	0.012
Loan repayment period				− 0.8612572	− 1.60	0.110
Financial report				1.588894	3.33	0.001
Lending procedure				0.3281761	0.69	0.491
Formal registration				0.5670824	1.46	0.144
Business plan				0.9532479	1.99	0.047
Location	Assosa Wereda as a reference					
Bambasi				1.047531	1.81	0.071
Menge				0.7714974	1.21	0.225
Homosha				0.2143832	0.26	0.798
Number of institutions				0.0998386	0.21	0.832
Inverse mills ratio				− 2.077474	− 1.27	0.203
	Number of obs = 296 LR chi2(13) = 70.10 Prob > chi2 = 0.0000 Pseudo R2 = 0.1719			Number of obs = 162 LR chi2(22) = 132.41 Prob > chi2 = 0.0000 Pseudo R2 = 0.6200		

## Abbreviations

MSSEs: Micro- and small-scale enterprises
MFIs: Micro-Finance Institutions
NBE: National Bank of Ethiopia
POH: Pecking order hypothesis
TVET: Technical and Vocational Education and Training
MoUDH: Minsitry of Urban Develeopment and Housing
